# Ensemble of RNN Classifiers for Activity Detection Using a Smartphone and Supporting Nodes

**DOI:** 10.3390/s22239451

**Published:** 2022-12-03

**Authors:** Marcin Bernaś, Bartłomiej Płaczek, Marcin Lewandowski

**Affiliations:** 1 Department of Computer Science and Automatics, University of Bielsko-Biała, Willowa 2, 43-309 Bielsko-Biała, Poland; 2 Institute of Computer Science, University of Silesia, Będzińska 39, 41-200 Sosnowiec, Poland

**Keywords:** mobile phone, sensor nodes, activity recognition, transmission suppression, recurrent neural network, classification ensemble

## Abstract

Nowadays, sensor-equipped mobile devices allow us to detect basic daily activities accurately. However, the accuracy of the existing activity recognition methods decreases rapidly if the set of activities is extended and includes training routines, such as squats, jumps, or arm swings. Thus, this paper proposes a model of a personal area network with a smartphone (as a main node) and supporting sensor nodes that deliver additional data to increase activity-recognition accuracy. The introduced personal area sensor network takes advantage of the information from multiple sensor nodes attached to different parts of the human body. In this scheme, nodes process their sensor readings locally with the use of recurrent neural networks (RNNs) to categorize the activities. Then, the main node collects results from supporting sensor nodes and performs a final activity recognition run based on a weighted voting procedure. In order to save energy and extend the network’s lifetime, sensor nodes report their local results only for specific types of recognized activity. The presented method was evaluated during experiments with sensor nodes attached to the waist, chest, leg, and arm. The results obtained for a set of eight activities show that the proposed approach achieves higher recognition accuracy when compared with the existing methods. Based on the experimental results, the optimal configuration of the sensor nodes was determined to maximize the activity-recognition accuracy and reduce the number of transmissions from supporting sensor nodes.

## 1. Introduction

Nowadays, sensor-equipped mobile devices have become a part of human life. Smartphones are still the most popular devices, despite newly created gadgets such as intelligent wear (e.g., trousers or jackets) or items such as rings and wristbands. In 2021, five billion smartphones with multiple sensors were enabled to measure the daily routines of their owners and recognize human activities. The scale of applicability of smartphones allows for collecting data to study human behavior and to influence human habits for better health. Mobile devices are also helpful for observing long-term characteristics that can be used to assess human populations’ health-related risk factors. Researchers have already demonstrated the usefulness of smartphones in monitoring physical fitness and recognizing health problems such as obesity, diabetes, various cardiovascular diseases, and even mental health [1,2,3,4,5]. Budget smartphones have sufficient storage, computing power, and transmission capabilities to become a powerful self-sufficient tool for human activity type classification [6]. However, there are several limitations that those devices have. The first one is related to the influence of device location and type of holder on sensor readings [6,7]. The second is a device’s energy utilization, which forces a user to turn off the sensing capabilities [6,8]. Finally, the mobile device is not always present with the user (e.g., stored in a bag or left at the office). Therefore, additional wearable sensor devices (as supporting nodes) are used within personal area networks (PANs) to mitigate this disadvantage [8]. The PAN network consists of multiple wearable sensors, usually connected via clothing or bands to the user’s body. The sensors collect, process, and transmit additional data via low-cost wireless communication to the main node [9,10]. The most common issue with a PAN network is the necessity of recharging the wireless sensor nodes. Thus, new solutions are required for reducing data transmission in PAN to minimize power consumption and extend the lifetime of the sensor nodes [11,12]. Previous research shows that the number of data transmissions can be significantly reduced by using intelligent wearable sensor nodes that process their sensor readings locally [8].

In this paper, a PAN is proposed which connects a smartphone with additional wearable sensor nodes to improve activity-recognition accuracy. An essential element of this solution is an algorithm of sensor nodes’ operation, which reduces the number of transmissions to extend the network lifetime. According to the proposed approach, the smartphone and its supporting sensor nodes perform activity classification independently with the use of recurrent neural networks (RNNs). The results of activity classification are then sent from supporting nodes to the smartphone if necessary. As the main sensor node, the smartphone makes a final activity assessment according to a voting procedure. This method was designed to recognize an extended set of human activities, which includes basic activities such as standing, lying, sitting, walking, and jogging; and exercises such as jumping, squatting, and star jumps. During experiments, the recognition accuracy was determined for eight activity classes. The objective of this research was also to analyze the impact of sensor node location on the accuracy of activity recognition.

The research reported in this paper is also focused on a new design for an RNN ensemble for distributed in-network activity classification. The individual RNN classifiers are introduced for particular sensor nodes to enable independent activity recognition based on local sensor readings. The local classification results determined by RNNs are selectively transmitted to the main node (smartphone). It should be noted that in this scheme, unnecessary data transmissions are suppressed. To this end, the RNN ensemble was integrated with a suppression mechanism which allows the supporting sensor nodes to decide if their current classification results are useful and should be reported to the main node. Moreover, a modified voting procedure was proposed for the main sensor node to aggregate the local classification results that can be reported by a subset of the supporting nodes.

The main research contribution of this work is a PAN that recognizes complex human activities by using a smartphone as a cluster head and additional wireless devices as the supporting sensor nodes. The main contributions of this paper are summarized as follows:

- A new deep-learning-based ensemble algorithm with event-type-driven suppression for activity recognition,
- Determination of the optimal device position for activity recognition,
- An extended set of recognized human activities, including non-standard ones,
- Analysis of various configurations of phone and sensors’ positions.

The results obtained from the analysis of various sensor node configurations allowed us to select the optimal set of sensor nodes and their positions. The feasibility and effectiveness of the proposed approach were confirmed in experiments with phone-based sensor nodes.

The paper is organized as follows. Section 2 reviews the works related to the applications of smartphones and wearable sensors in human activity recognition. Section 3 presents the proposed model for activity recognition with smartphone and wearable sensors. This model was verified by experiments, as described in Section 4. Finally, the discussion of the experimental results and conclusions are included in Section 5.

## 2. Related Works

In the literature, various human activity recognition (HAR) methods were proposed that are based on various classification algorithms. The objective of HAR is to recognize current human activity by taking into account sensor measurements, e.g., acceleration, rotation speed, magnetic field changes, or geographical coordinates. Usually, the recognition is based on measurements collected in a given period of time. Most existing HAR approaches involve data acquisition, pre-processing, feature extraction, and classification [6].

### 2.1. Data Acquisition and Pre-Processing

Sensor data are usually acquired for a given group of activities. Most of the up-to-date research works focus on a small set of basic activities such as sitting, standing, walking, or running. There are several works that consider different activities, e.g., normal and fast walking [13], training activities [14], or common house activities [15]. More recent studies are devoted to recognizing a single type of activity, e.g., walking [16]. In [17], a set of activities was considered which is specific for physical workers. The authors of [18] have analyzed the activities by taking into consideration the physical attributes of individual persons, such as height, weight, and gender. The research results have shown that a model’s adaptation to personal physical characteristics can significantly improve the recognition result. Despite various proposals, the comparative study in [7] has demonstrated that no single HAR procedure can work well for various activities in all settings. Those results highlight the importance of designing new HAR methods and algorithms that address specific types of activities. In this research, we extended the set of activities that are considered in the related literature.

The selection of sensors depends on the set of recognized activities. Typical solutions utilize data from an accelerometer, a gyroscope, a magnetometer, GPS, a proximity sensor, and a light sensor. Additionally, other data sources can be used that indirectly allow us to classify a given activity, i.e., ambient pressure, humidity, and temperature. The available hardware platform usually limits the selection of sensors. The most common hardware platforms [6] allow us to use an accelerometer, gyroscope, and magnetometer to capture body movements. In the case of mobility activities, the barometer and GPS sensor are also useful [19]. Some previous research works were devoted to recognizing location or activity using illumination sensors [20]. In this research, a solution is proposed for implementation with the majority of smartphone models. Thus, only the most popular sensors, available for a broad spectrum of smartphones, were considered, i.e., the accelerometer and gyroscope.

Another crucial issue concerns sampling frequency, which significantly influences classification accuracy and energy consumption. The previous studies show that data collected from an accelerometer and gyroscope at a 10 Hz sampling rate are sufficient to distinguish between various types of simple activities [21]. The sampling rate reduction can significantly increase a device’s lifetime [22]. The sampling issues can be resolved using up-sampling or down-sampling of data [23,24]. An equally important aspect is the organization of the acquisition process and the device’s location on the human body. In real life, each smartphone user carries the device differently, impacting the accelerometer, gyroscope, and magnetometer readings. On the one hand, the results of research [25] suggest that HAR algorithms robust to the position deviations of the smartphone can be designed using the selected sensors. On the other hand, several research works show the influence of different smartphone positions on the recognition accuracy, i.e., in jacket [26] or in hand [27].

The above-mentioned sampling and placement issues are also analyzed in this work for an extended set of activities.

### 2.2. Feature Extraction and Classification

The state-of-the-art HAR methods in the literature use various feature extraction and classification approaches, where data relations are found using statistics, visualizations, or learned as a part of the machine learning process (usually based on neural networks) [6,28]. In [25], it was presented that frequency spectrum analysis can give promising results for recognizing movement activities. The features for classification are extracted as statistics (e.g., local mean, variance, absolute deviation, correlation, or gradients) for a given time window. The previous research results show that recognition accuracy can be improved by increasing the width of the time window length [28,29,30]. Some research was also conducted to pick up one repeating pattern of a particular activity, such as walking or running [31]. Recently, the features were learned using deep neural networks, where data were processed as raw time series [32] or feature vectors in the time or frequency domain [33]. That method uses a convolution layer to select the specific features for a given activity. Several, up to tens of features can be selected as input data for the activity classification algorithm when using the above-mentioned methods.

A classification model is necessary to recognize human activities. Usually, the classification model is obtained via supervised learning based method on selected features or a raw dataset with labels representing the actual activities. Multiple classifiers were used for this purpose, from simple algorithms such as *k*-nearest neighbors [34], support vector machine [35], or naive Bayes [36] to the ensemble classifiers, such as random forest [37], AdaBoost [18], or deep neural networks [38,39]. Previous studies have shown that simple classifiers can give satisfactory results (overall accuracy of over 95%) in the case of simple activities [34,35,36]. However, the experimental results in [6] show that ensemble classifiers tend to outperform single classifiers, and the classifiers based on deep learning tend to outperform the above types.

Deep learning with recurrent neural architecture has become a popular solution to tackle time series data from sensors. The authors of [40] have proposed an RNN to overcome the long-range dependencies problem in sequences. They proved the high efficiency of the method for LSTM RNN networks using several datasets. A stacked LSTM network for activity recognition consisting of five LSTM cells was proposed in [41]. In that solution, the sensor data are pre-processed using a one-layer network. The network at the training stage uses an $L_{2}$ regularizer in the cost function to improve model generalization, which improves the solution’s accuracy. LSTM was used with success in [42] for modeling spatio-temporal sequences obtained by smart sensors. The experimental results demonstrate the advantages of using LSTM over other machine learning approaches. Similar results were obtained for HAR by applying two-layer LSTM with convolutional layers [43]. The experiments on publicly available datasets have confirmed an accuracy of over 95% for basic activity types. Recent research shows that the gated recurrent units and the long-short term memory technique achieve the best accuracy and training time [44]. The analyzed solutions allow us to obtain high accuracy (over 95%) when tackling typical activities. However, as was presented in [45], there is still a lack of a unified human activity model for wearable sensors. The research results have shown that the numbers and positions of sensors in the existing wearable HAR systems vary significantly, which affects their promotion and application. The experiments reported in [45] have proved that having the appropriate locations for a smaller number of sensors can enable the same classification effect. Accuracy of over 90% was achieved for locomotion and gesture activities. Finally, the ensembles of deep neural networks (or ensembles of classical models) show viable solutions for multiple activity recognition [46].

In this paper, the impact of smartphone location is analyzed in the context of deep learning methods. Furthermore, the tradeoff between recognition accuracy and energy consumption is taken into consideration.

### 2.3. Transmission Using Additional Nodes

Most existing approaches to HAR use a single sensing device (e.g., a smartphone). However, one device could be insufficient for the recognition of more complex activities. Thus, combinations of several devices were considered in the literature to increase HAR accuracy [8,9,10]. In this case, additional issues arise concerning data transmission between devices. It should be noted that we consider only direct one-hop communication, where a smartphone serves as a cluster head [9]. In this scenario, the number of transmissions and power consumption can be reduced by data aggregation [47], elimination of repeated overhead transmission, or pattern analysis [48]. The data can be transmitted as raw sensor readings or transformed into more complex structures such as graphs [49]. Another approach, called prediction-based suppression, allows the device to decide if data should be sent to a sink and updates the current inputs of a prediction model [50].

Other related methods are based on the event-trigger machine learning approach, where data are sent only if an event is detected [51]. In [8], the authors proposed an approach where the classifier decides if data should be sent to the sink for HAR purposes. The HAR system presented in this paper combines the methods in [8] and [51]. According to the proposed approach, the local result of activity classification is sent to the smartphone only if it is needed for activity recognition.

## 3. Proposed Method

**Figure 1:**
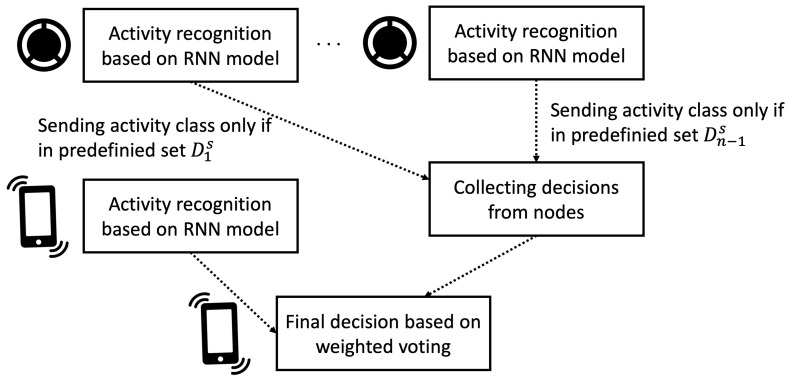
Overview of the proposed personal area network model for activity classification.

The proposed PAN includes a mobile device (smartphone), which is used as the main node (cluster head) and collects classification results from the supporting sensor nodes. After gathering information from supporting nodes, the main node makes the final decision based on the ensemble classification approach. Its objective is to recognize a person’s activity at the current time step (*t*). The supporting nodes are only used to recognize more complex activities. Thus, these nodes transmit their classification results to the cluster head if the results are necessary for recognizing current activity. The proposed approach is based on the observation in [6] that most simple activities can be detected with high accuracy using one node only. For most complex activities, additional nodes are required. In contrast to the method proposed in [8], where raw sensor data are transmitted, in the case of the proposed approach, the result of local classification is sent if it can influence the outcome of the ensemble classification. The overview of the proposed model is presented in Figure 1.

The decision about the necessity of sending the classification result ($d_{i,t}$) at time step *t* by node *i* is made using the following formula:

(1) $$s_{i,t}=\left\{\begin{array}{cc} 1 & d_{i,t}\in D_{i}^{s}\\ 0 & d_{i,t}\notin D_{i}^{s} \end{array}\right.$$

where: $d_{i,t}$ is an activity recognized by sensor node *i* at time step *t* and $D_{i}^{s}$ denotes the set of activity classes for which sensor node *i* has to transmit data.

The sensor node *i* sends $d_{i,t}$ to the main node only if $s_{i,t}=1$. Intuitively, some activities consist of the specific arm or leg movements that are important for correct activity recognition. Thus, for each sensor node location, a separate set $D_{i}^{s}$ is selected using optimal set search. The set of activity classes $D_{i}^{s}$ is a subset of set *D*, which includes all activity classes under consideration. The activity classes are denoted by natural numbers, e.g., $D=\left\{1,2,3,4,5\right\},D_{i}^{s}=\left\{1,3,5\right\}$. The set $D_{i}^{s}$ is found at the training phase (as described in Section 3.1). It contains only those activity classes for which the smartphone cannot correctly recognize the activity based on its own sensor readings.

The classification result $d_{i,t}$ is determined by the *i*-th node using a machine learning model $M_{i}$ and a set of sensor readings $X_{i}\left(t\right)$. Although various machine learning techniques can be used to create classification models for a given dataset, the deep learning approach was selected for HAR [6] as the most promising one. Its specification is described in Section 3.2. The result of classification obtained by sensor node *i* includes the activity class $(d_{i,t}$ and its weight $\left(w\left(d_{i,t}\right)\right)$. The final ensemble classification is performed by the main node using a voting procedure. In the voting procedure, the weights of local classification results are taken under consideration as described in the following formula:

(2) $$d_{t}=argmax_{j=1..card(D)}\left(\sum_{d_{i,t=j,i=1..n}}w\left(d_{i,t}\right)\right)$$

where: *j* denotes the activity class, *w* is the weight of the activity class, card(D) is the cardinality of the activity set, and *n* corresponds to the number of sensor nodes, including the main node (smartphone).

The smartphone acts as the main sensor node by executing two independent operations in parallel within a time window of width dt:

Data collection:

- Receive results of local classification $d_{i,t}$ and $w(d_{i,t})$ from supporting sensor nodes.

Data processing:

- If t>0, calculate the final recognition result $d_{t-1}$ according to Equation (2).
- Determine $d_{n,t}$ based on its own sensor readings.

**Figure 2:**
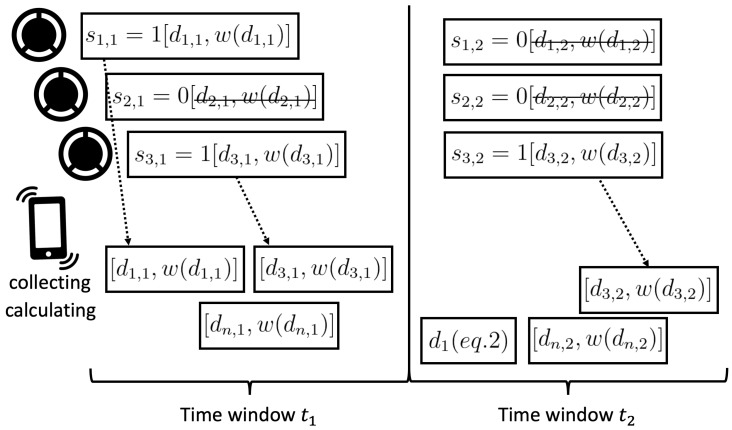
Cooperation of sensor nodes in proposed personal area network.

This approach requires the data collection and data processing operations to be performed simultaneously on the main node, as presented in Figure 2.

The scheme in Figure 2 presents the interaction between the sensor nodes within the considered PAN. The individual classification results of supporting sensor nodes with their weights allow the main node to make the final assessment in accordance with Equation (2). It is worth noting that this approach is robust to the failure of a supporting sensor node. The presented solution enables activity recognition even when no additional data from supporting sensor nodes are provided. However, in such a case, the recognition accuracy may be decreased. The required size of the time window dt depends on the number of supporting nodes and transmissions that are necessary to recognize the considered activities.

### 3.1. Optimal Activity Set Finding for a Supporting Sensor Node

The number of transmissions is strictly correlated with the energy usage of sensor nodes. This issue is especially important in the case of supporting nodes because the users are reluctant to use additional devices if they have to be recharged very often [6]. Therefore, the presented algorithm aims to reduce the transmissions only to those activities that cannot be precisely detected by the main node (smartphone). Therefore, the optimization strategy seeks to minimize the size of set $D_{i}^{s}\subseteq D$, for each supporting node (*i*), provided that the overall accuracy of activity recognition does not decrease. The optimization is performed according to the following procedure (Algorithm 1):
**Algorithm 1** Optimization strategy for reducing the set of transmitted data.1:Set $D{}_{i}^{s}=D$, for all nodes i=1,...,n, *n* is designated to smartphone2:Calculate accuracy *a* based-on training set, $D_{i}^{s},i=1...n$ and Equation (2)3:**for each** i=0...n−1 **do**4:    **for each** activity *j* in $D_{i}^{s}$ **do**5:        Calculate accuracy $a_{i,j}$ based on training set and $D_{i}^{s}-j$ and Equation (2)6:        **if** $a-a_{i,j}\leq \varepsilon$ **then**7:           remove activity *j* from $D_{i}^{s}$ set and go to 28:        **end if**9:    **end for**10:**end for**

Algorithm 1 starts with recognizing the activities based on information from all sensor nodes. Then, it calculates the activity-recognition accuracy. The same operation is performed in the next step for a set without data related to one activity class (j) from one supporting sensor node (i). If the decrease in recognition accuracy is not larger than ϵ, then the data are removed from $D_{i}^{s}$, and the optimization continues. The new set $D_{i}^{s}$ is treated as a baseline for further optimization. The parameter ϵ is introduced to eliminate the influence of outliers on the optimization result. Its value should be low, i.e., ϵ=0.001. The above optimization method is based on an exhaustive search due to the limited number of supporting sensor nodes and activities. It should be noted that the main node performs the classification at each time step to ensure that the recognition result is obtained even if no supporting node reports its local classification result.

### 3.2. RNN Model Construction

**Figure 3:**
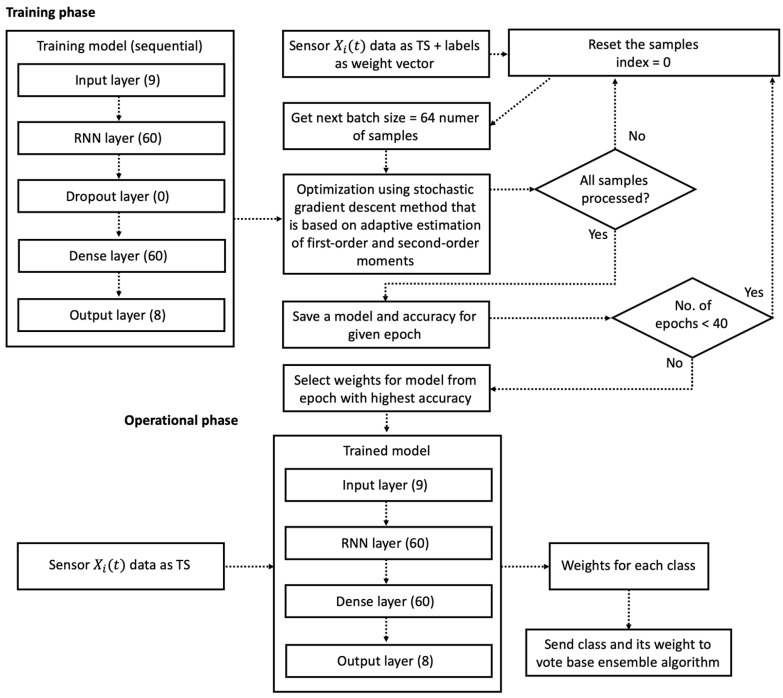
Training and operation of the RNN model for activity recognition.

The RNN classifier was selected based on a review of the literature [40,52]. Its implementation uses long short-term memory cells (LSTMs). Compared to a classical neural network, this network allows us to perform classification based on raw time series (TS). In the proposed approach, the time series include accelerometer and gyroscope data readings. These sensor readings are fed directly into the neural network for training. The training procedure creates the model for activity recognition. An additional advantage of this model is that it can process sequential data. The time series are divided into sequences using fixed-width sliding windows of *s* readings (s=128 by default [52]). The gravitational and body-motion components were separated in the accelerometer signal using an incremental filter. Thus, the gravity effect was filtered out of the accelerometer readings. The constructed RNN model takes many input vectors to process them and provides one output vector. Therefore, a so-called "many to one" architecture is used: we accept time series of feature vectors (one vector per time step) to convert them to a probability vector at the output for classification. The model training procedure and its usage are presented in Figure 3.

The RNN’s internal structure and the number of neurons in each layer (defined in parentheses in Figure 3) were adopted from [40,52], but the tuning process and the number of preprocessing operations were modified. The number of epochs was reduced, and an additional dropout layer was added to avoid model overtraining and to obtain weights which could be used in the voting procedure. The activity with the highest weight is recognized as an actual activity. The modified parameters used in this research are presented in Listing 1 with the TensorFlow Keras [53] script.

TensorFlow [53] is a programming library that allows us to define and implement various machine learning algorithms. The advantage of TensorFlow is that it supports heterogeneous systems. Thus, it is possible to train a model on systems with GPU support and then use it on mobile phones without modifications. The library in version 2.0 supports various deep neural network models and thus has been used in this research to implement the machine learning methods.

During the RNN’s training, the initial value of a batch size was selected based on the settings proposed in [52]. These settings allow us to reduce the memory utilization of the workstation, as presented in Figure 3. The number of epochs was determined experimentally to avoid overtraining of the neural network, which is especially harmful in the case of the proposed voting procedure. Detailed information regarding the maximal number of epochs is presented in Section 4.

The training procedure is based on the replacement optimization algorithm for stochastic gradient descent (Adam). This algorithm has polynomial computation complexity [54]. However, its training efficiency allows us to decrease the number of epochs in the training process. It should be noted that the training process is performed using a workstation with high computational power. In contrast, the classification procedure can be executed on mobile devices using the trained model with linear complexity.
**Listing 1** TensorFlow Keras script for RNN implementation1:verbose = 0                                                                                                                 ▹ data presentation off2:epochs = 40                                                                                                     ▹ maximal number of epochs3:batch_size = 64       ▹ number of samples that will be propagated through the network4:n_timesteps, n_features, n_outputs = trainX.shape[1], trainX.shape[2], trainy.shape[1]5:model = Sequential()6:model.add(LSTM(60, input_shape=(n_timesteps,n_features)))7:model.add(Dropout(0.5))8:model.add(Dense(60, activation=’relu’))9:model.add(Dense(n_outputs, activation=’softmax’))10:model.compile(loss=’categorical_crossentropy’,                   optimizer=’adam’,                   metrics=[’accuracy’])

## 4. Experiments and Discussion

The experiments were conducted to evaluate the effectiveness of the proposed activity recognition method and to demonstrate the benefits of using additional sensor nodes that support the main node (smartphone). During experiments, the criteria of recognition accuracy and transmission reduction were considered to verify if the proposed method enables accurate activity recognition and contributes to energy savings of the supporting nodes. At first, the optimal position of a smartphone was determined for daily activities, and then the set of activities was extended by fitness exercises. Finally, the influence of additional sensors on activity-recognition accuracy was investigated. The results obtained for the proposed method were compared with the state-of-the-art methods [6].

### 4.1. Experimental Testbed

**Figure 4:**
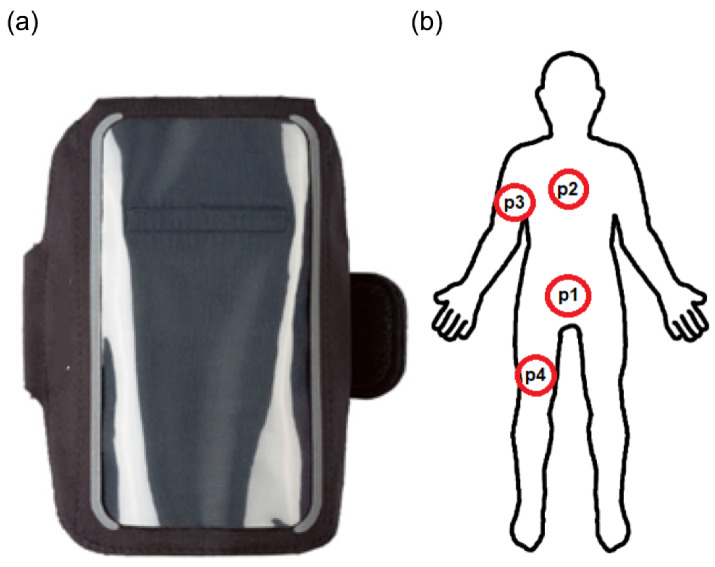
Elements of experimental testbed: (**a**) holding strap, (**b**) smartphone positions.

The Realme 8 5G smartphones were used as the main sensor node and the supporting sensor nodes. The device was selected as medium-level equipment that represents hardware platforms of average cost. The smartphone is equipped with an ARM Dimensity 700 Octa-core processor with 6 GB of RAM, allowing it to process data and perform recognition efficiently. The device has accelerometer and gyroscope sensors that enable tracking the user’s movement. The device can communicate via Bluetooth 5.0, Wi-Fi ver. 5, and GSM. It is worth noting that, as in low-cost devices, the Bluetooth and Wi-Fi interfaces share the same antenna; thus, for the time of experiments, Wi-Fi was disabled. The smartphone was attached using the sport band in several places during experiments. The type of phone holder and placement positions are presented in Figure 4.

The mobile devices were attached to various body parts (waist, chest, arm, and upper leg) using the holding strap. In the initial research, the additional sensors for ankles and wrists were considered. However, during experiments, they strongly influenced the movement. Moreover, in those places, the smartphone cannot be held comfortably. Thus, this possibility was excluded from further research. The data from sensors were gathered in 2 min training sessions, which were repeated multiple times. The classification models were trained and verified using Waikato Environment for Knowledge Analysis (WEKA package) and Konstanz Information Miner (KNIME) software [55]. The RNN implementation was prepared using the TensorFlow library. These environments together allow us to analyze hundreds of configurations and their variants. As mentioned above, the data collection was conducted using the devices for intervals of 2 min. The time series were registered with a 10 ms sampling rate and sent to a database. The activity was registered continuously for the given period. The first and last two percent of the time series were excluded from processing to remove the preparation and stop phase of the activity. The considered activities include:

- walking at a normal pace, where legs and arms are moving without any luggage,
- jogging at a moderate pace, where limb movement is faster and the body is moving more dynamically,
- squats, where a person is performing squats in place with arms maintaining balance,
- jump, where a person jumps in place as a part of aerobic exercise,
- lying, where a person is lying on his back, and major movement corresponds to chest breathing,
- arms swing, where the arms are moving during the exercise or housework,
- siting, where the person sits at the desk and performs simple writing (office) activities,
- standing in place, where only natural body balance can be registered.

### 4.2. Results and Discussion

**Figure 5:**
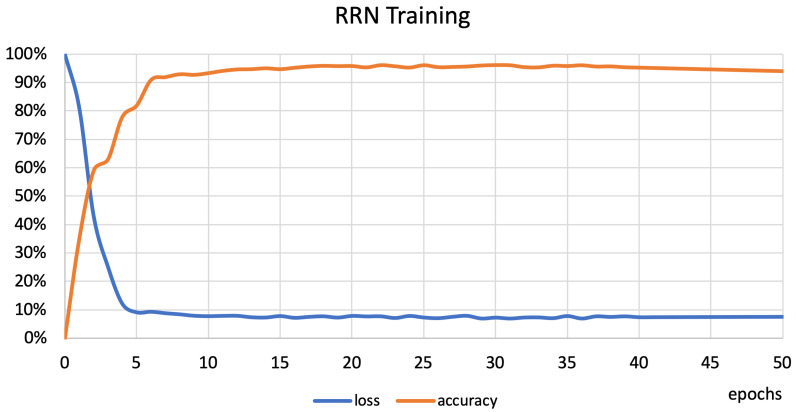
Training of the RNN classifier.

The first experiment was conducted to find the sensor frequency at which the classification results are stable and the minimal number of readings is required. The set of activities was divided into two subsets: basic activities such as walking, jogging, or sitting; and extended activities such as fitness exercises. The research concerning basic activities was conducted to confirm that the activities considered in previous works [6,8,9,10,11,12,13] can be recognized at the presented testbed. The data were divided into training and verification sets. Firstly, the stability of the proposed classifier was verified. Figure 5 shows the training curve of the neural network (RNN). This figure shows that the accuracy increases rapidly to the 5th epoch, then stabilizes around the 20th epoch, and increases slowly up to the 40th epoch. Then, it starts to decrease slowly to the 50th epoch. Therefore, the maximum number of epochs was set to 40. The results were analyzed for the phone beig held on the waist, chest, leg, and arm. Each RNN was trained ten times to obtain stable results.

**Figure 6:**
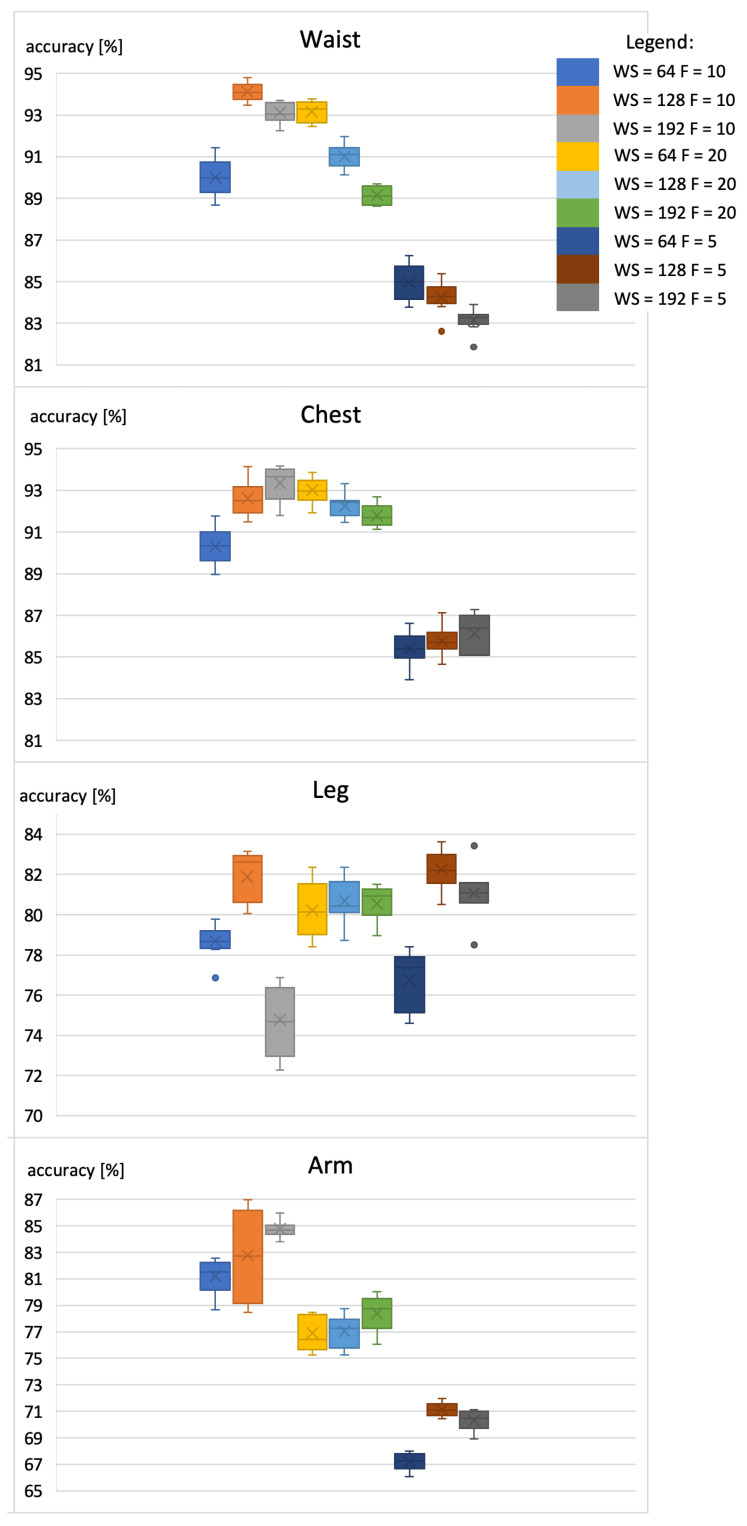
Accuracy of standard activities recognition using the smartphone with the RNN classifier.

The accuracy of basic activity recognition was determined for different settings of two parameters: the interval between the sensor readings (F) and window size (WS). The results are presented in Figure 6. As proposed in [6], the common WS equals 64, 128, or 196 samples per evaluation; and *F* equals 5, 10, or 20 Hz.

**Figure 7:**
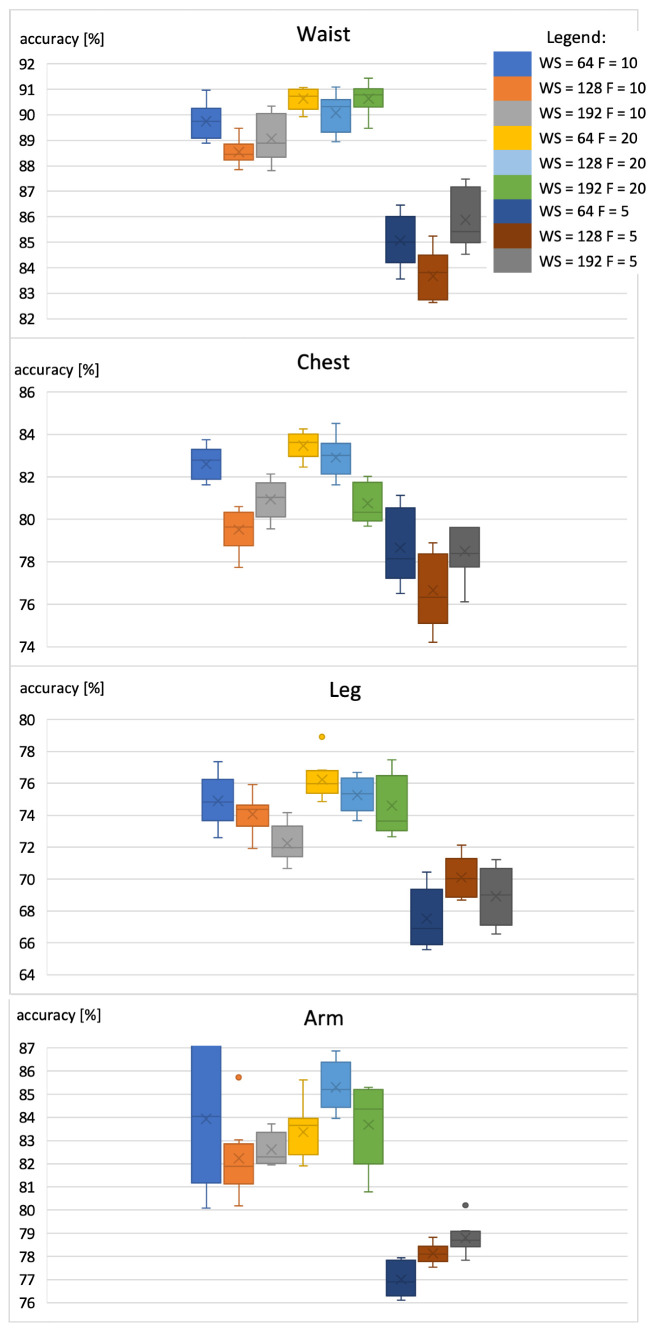
Accuracy of extended activity recognition using the smartphone with the RNN classifier.

The results presented in Figure 6 confirm the method’s high accuracy in classic activity recognition. As was presented in [40,52], high accuracy (almost 95%) was obtained using a smartphone attached to the waist. The best and most stable result was achieved in the case when WS equals 128 samples, and the sample rate was set to 10 Hz. The results obtained for different window sizes show that enlarging the window above 128 samples will not affect the accuracy. Moreover, the research results show that the chest is also a good place to hold a phone when performing activity recognition. It decreases the detection accuracy in daily routines only by 1%. The most stable solutions were achieved for the window size equal to 128 samples. It is even possible to increase the accuracy of this model further, however, at the cost of its stability. The result confirms that sample rate of 10 Hz is sufficient for recognizing the basic activities. The increased sampling rate of 20 Hz did not influence the activity-recognition accuracy. The analogical research was conducted for the extended set of activities, including training activities such as jumping, squats, and arm swinging. The results are presented in Figure 7.

The results in Figure 7 show that when extending the set of activities, the accuracy for the smartphone placed on the waist, chest, and leg decreased, respectively, by 5, 10, and 6%. However, the recognition accuracy for the device placed on the arm increased by about 1%. This change was caused by the fact that for the two new activity classes, high recognition accuracy was obtained using the sensors placed on the arm. What is also interesting is that the results for WS of 64 samples are comparable to those obtained with WS of 128 samples. Additional experiments were conducted to verify the results for the window size of 32 samples. However, the accuracy was significantly lower than that obtained for WS of 64 samples. This could be caused by the type of activity, where the movement repeats within 1 second. Moreover, by increasing the sampling frequency to 20Hz, it is possible to obtain better results in the case of the extended set of activities. Therefore, the parameters WS = 64 and F = 20 Hz were set for the next experiment. The selected parameters were used to evaluate the influence of the main sensor node’s (smartphone) position on the detection accuracy.

The tests were performed for the ensemble classification method presented in Section 3. In total, 16,136 samples were used in this experiment. The classifier was trained using 80% of the data and then verified using the test set (20%). The results are presented in Table 1, where the 1s in the vectors mean that the supporting sensor node will transmit the classification result if a given activity is recognized.

The results in Table 1 firmly show that it is possible to increase the accuracy of activity recognition by using the sensor network with supporting nodes. The highest accuracy for the single node is achieved when the phone is placed on the waist. However, using at least two sensor nodes makes it possible to increase the recognition accuracy by 2% compared to the solution with a single node. Table 1 shows the best positions of sensors, with their configuration. The most promising sets of nodes are $s_{1}$ and $s_{3}$, and $s_{2}$ and $s_{3}$. For the presented sets, the first sensor acts as the main node and the rest as support nodes. In the case of using three nodes, the best configurations include $s_{1}$ with $s_{2}$ and $s_{3}$; and $s_{2}$ with $s_{1}$, $s_{3}$. It is worth noting that the sets $s_{3}$ with $s_{1}$ and $s_{2}$; and $s_{4}$ with $s_{1}$ and $s_{2}$ were also promising at the training stage. Moreover, all configurations showed a significant increase at this stage. Thus, all configurations were verified. The results of testing the recognition accuracy and transmission suppression are presented in Table 2.

**Figure 8:**
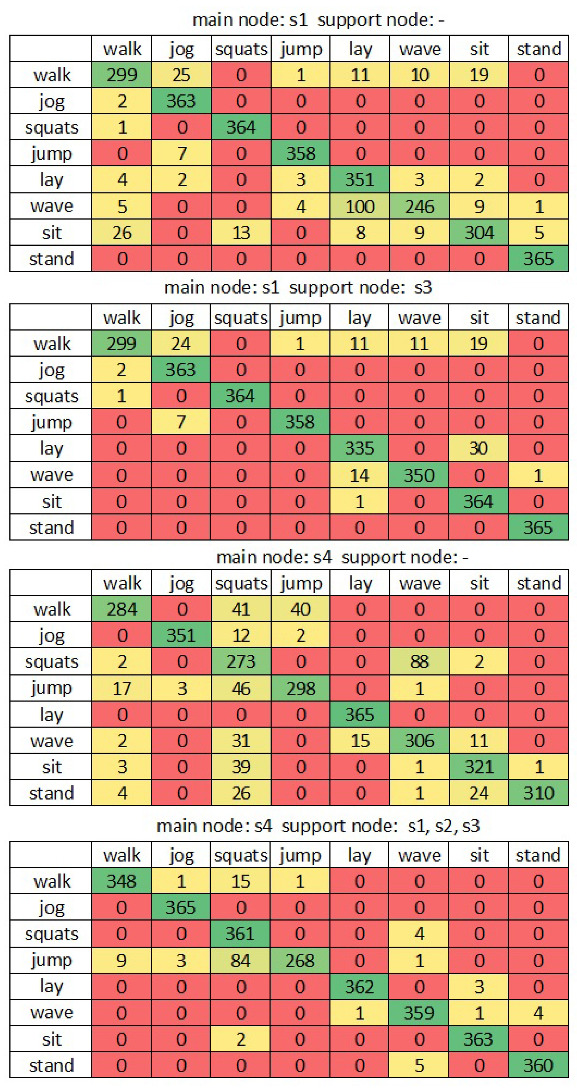
Confusion matrices for various sensor node configurations.

The results in Table 2 show that a single node allows us to detect the basic activities and more complex activities with almost 91% accuracy. When one supporting node is added (placed on a chest or leg), it is possible to increase the accuracy to 96% for the given dataset. The research results show that the main sensor placed on the chest is the least efficient. However, this placement is useful for the supporting node. The best results, in terms of accuracy and data suppression, were obtained for the pair $s_{1}$ and $s_{3}$, which achieved suppression of 50%. An accuracy close to 99% can be achieved when using three nodes. However, the accuracy and number of transmissions are not significantly improved. The best result can be achieved in the case of four nodes, where the waist node is the main one. For this configuration, the accuracy above 99.5% was achieved with 58% suppression. The confusion matrices in Figure 8 present the misclassifications between particular activities.

The sensor node placed on the waist had the most significant difficulties in distinguishing training activities. This was caused by the fact that the ongoing activities have similar moving pattern. By using the on-leg sensors, this issue was solved. Only the training routine was misclassified as walking. The same situation was the case for sitting and standing for the sensor placed on the arm. Accuracy of 95.4% can be achieved for the extended dataset when using the proposed procedure with all sensor nodes and the arm as a main sensor node. If the main sensor node is on the waist, the accuracy increases to 99.5%. The smartphone accurately performs the recognition of basic activities. Thus, the recognition has to be supported by additional nodes only in case of activities with similar movement patterns registered in main node. This observation allowed us to reduce the number of transmissions from supporting nodes by 63%. This suppression level can be achieved when activity duration is balanced. In practice, when training activities are less often than regular activities, the number of needed transmissions can be even lower.

## 5. Conclusions

The paper introduced a PAN for human activity recognition. The proposed method allowed us to extend the activity recognition capabilities of a smartphone by using supporting sensor nodes such as bands or wearable sensors. The method is based on a deep learning approach and an ensemble classifier with RNNs. It was demonstrated that the method suppresses unnecessary transmissions and reduces the energy consumption of the supporting nodes. The potential energy savings are essential in practice, as they enable the longer use of supporting nodes without recharging. The experimental results show that the proposed approach increases the activity-recognition accuracy by 8% and reduces the number of data transmissions by 58% (without losing accuracy), which means that the lifetime of supporting nodes can be significantly extended. The proposed method can be further enhanced by modifying the ϵ parameter in algorithm 1 to find a balance between accuracy value and suppression value.

Additionally, the research results show that the optimal sampling rate for the supporting node is 20 Hz and window size equals 64 samples. A lower sampling rate also increases the lifetime of all sensor nodes. It was demonstrated that for activity recognition, the best placements of the main and the supporting sensor nodes are on the waist and the leg or chest, respectively. For this configuration, one can achieve an accuracy of activity recognition above 96%. When additional sensor nodes are introduced, the accuracy can be increased to 98% with two supporting nodes and to 99.5% for three supporting nodes.

The potential directions of future research include the following:

- The development of recognition methods that enable movement detection of single and multiple body parts to categorize them and divide them into activity groups. The optimal set of sensors (with their position) will be proposed based on such methods for each activity group and their combinations.
- The combination of different sensor modalities aimed to resolve the issue of noisy data from wearable sensors. The potential research can establish the background for a broader range of recognized activities, e.g., using vision sensors.
- The development of more advanced data suppression methods to further reduce the transmitted data from supporting sensor nodes and to increase their lifetime. Additionally, other suppression methods, as presented in [8], could be adopted.

## Figures and Tables

**Table 1:** Training accuracy obtained for the proposed method using the network with supporting nodes.

Main Node	Support Node	$D_{i}^{s}$ Vectors	Accuracy Based on Training Set [%]
$s_{1}$	$s_{2}$	[1, 1, 0, 1, 1, 1, 1, 0]	96.92
$s_{1}$	$s_{3}$	[1, 0, 0, 0, 1, 1, 1, 0]	97.12
$s_{1}$	$s_{4}$	[1, 1, 0, 1, 1, 1, 1, 0]	97.26
$s_{1}$	$s_{2}$, $s_{3}$	[1, 0, 0, 0, 0, 1, 0, 0], [1, 0, 0, 0, 1, 1, 1, 0]	99.86
$s_{1}$	$s_{2}$, $s_{4}$	[1, 0, 0, 0, 0, 1, 1, 0], [1, 1, 0, 1, 1, 1, 1, 0]	99.25
$s_{1}$	$s_{3}$, $s_{4}$	[1, 0, 0, 0, 0, 1, 1, 0], [1, 1, 0, 1, 1, 1, 1, 0]	99.45
$s_{1}$	$s_{2}$, $s_{3}$, $s_{4}$	[1, 0, 0, 0, 0, 0, 0, 0], [1, 0, 0, 0, 0, 1, 1, 0], [1, 1, 0, 1, 1, 1, 1, 0]	100.00
$s_{2}$	$s_{1}$	[1, 1, 0, 1, 1, 1, 0, 1]	87.05
$s_{2}$	$s_{3}$	[1, 0, 0, 1, 1, 1, 1, 1]	94.93
$s_{2}$	$s_{4}$	[1, 1, 0, 1, 1, 1, 1, 1]	86.92
$s_{2}$	$s_{1}$, $s_{3}$	[1, 1, 0, 1, 1, 0, 0, 0], [1, 1, 0, 1, 1, 1, 1, 1]	99.38
$s_{2}$	$s_{1}$, $s_{4}$	[0, 1, 0, 0, 0, 1, 0, 0], [1, 1, 0, 1, 1, 1, 1, 1]	96.71
$s_{2}$	$s_{3}$, $s_{4}$	[1, 1, 0, 0, 0, 1, 0, 0], [1, 1, 0, 1, 1, 1, 1, 1]	98.90
$s_{2}$	$s_{1}$, $s_{3}$, $s_{4}$	[0, 1, 0, 0, 0, 0, 0, 0], [1, 0, 0, 0, 0, 1, 0, 0], [1, 1, 0, 1, 1, 1, 1, 1]	99.86
$s_{3}$	$s_{1}$	[1, 1, 0, 1, 1, 1, 0, 0]	90.82
$s_{3}$	$s_{2}$	[1, 1, 0, 1, 1, 1, 1, 0]	93.63
$s_{3}$	$s_{4}$	[1, 1, 0, 1, 1, 1, 0, 0]	91.37
$s_{3}$	$s_{1}$, $s_{2}$	[0, 0, 0, 1, 1, 1, 0, 0], [1, 1, 0, 1, 1, 1, 1, 0]	99.18
$s_{3}$	$s_{1}$, $s_{4}$	[1, 1, 0, 0, 0, 1, 0, 0],[1, 1, 0, 1, 1, 1, 0, 0]	97.60
$s_{3}$	$s_{2}$, $s_{4}$	[1, 1, 0, 0, 0, 1, 0, 0], [1, 1, 0, 1, 1, 1, 0, 0]	98.49
$s_{3}$	$s_{1}$, $s_{2}$, $s_{4}$	[0, 0, 0, 0, 0, 0, 0, 0],[ 1, 1, 0, 0, 0, 1, 0, 0], [1, 1, 0, 1, 1, 1, 0, 0]	99.93
$s_{4}$	$s_{1}$	[1, 1, 1, 0, 0, 1, 1, 1]	95.62
$s_{4}$	$s_{2}$	[1, 1, 1, 0, 0, 1, 1, 1]	96.92
$s_{4}$	$s_{3}$	[1, 1, 1, 0, 0, 1, 1, 1]	93.77
$s_{4}$	$s_{1}$, $s_{2}$	[1, 1, 0, 0, 0, 0, 0, 0],[ 1, 1, 1, 0, 0, 1, 1, 1]	99.04
$s_{4}$	$s_{1}$, $s_{3}$	[1, 1, 0, 0, 0, 0, 0, 0], [1, 1, 1, 0, 0, 1, 1, 1]	97.33
$s_{4}$	$s_{2}$, $s_{3}$	[1, 1, 0, 0, 0, 0, 0, 0], [1, 1, 1, 0, 0, 1, 1, 1]	98.42
$s_{4}$	$s_{1}$, $s_{2}$, $s_{3}$	[1, 0, 0, 0, 0, 0, 0, 0], [1, 1, 0, 0, 0, 0, 0, 0], [1, 1, 1, 0, 0, 1, 1, 1]	99.93

legend: $s_{1}$—waist, $s_{2}$—chest, $s_{3}$—leg, $s_{4}$—arm.

**Table 2:** Verification of selected sensor node configurations.

Main Node	Support Node	Accuracy [%]	Suppression [%]
$s_{1}$	-	90.75	-
$s_{2}$	-	81.20	-
$s_{3}$	-	75.34	-
$s_{4}$	-	85.89	-
$s_{1}$	$s_{2}$	96.47	25
$s_{1}$	$s_{3}$	95.82	50
$s_{1}$	$s_{4}$	95.86	25
$s_{1}$	$s_{2}$, $s_{3}$	98.53	63
$s_{1}$	$s_{2}$, $s_{4}$	98.94	44
$s_{1}$	$s_{3}$, $s_{4}$	98.25	44
$s_{1}$	$s_{2}$, $s_{3}$, $s_{4}$	99.59	58
$s_{2}$	$s_{1}$	85.96	25
$s_{2}$	$s_{3}$	93.15	25
$s_{2}$	$s_{4}$	86.68	13
$s_{2}$	$s_{1}$, $s_{3}$	98.12	31
$s_{2}$	$s_{1}$, $s_{4}$	86.88	44
$s_{2}$	$s_{3}$, $s_{4}$	86.82	38
$s_{2}$	$s_{1}$, $s_{3}$, $s_{4}$	86.82	58
$s_{3}$	$s_{1}$	89.59	38
$s_{3}$	$s_{2}$	91.20	25
$s_{3}$	$s_{4}$	90.86	38
$s_{3}$	$s_{1}$, $s_{2}$	96.95	44
$s_{3}$	$s_{1}$, $s_{4}$	93.84	50
$s_{3}$	$s_{2}$, $s_{4}$,	95.48	50
$s_{3}$	$s_{1}$, $s_{2}$, $s_{4}$	95.48	67
$s_{4}$	$s_{1}$	92.64	25
$s_{4}$	$s_{2}$	85.00	25
$s_{4}$	$s_{3}$	90.82	25
$s_{4}$	$s_{1}$, $s_{2}$	85.68	50
$s_{4}$	$s_{1}$, $s_{3}$	93.18	50
$s_{4}$	$s_{2}$, $s_{3}$	94.66	50
$s_{4}$	$s_{1}$, $s_{2}$, $s_{3}$	95.41	63

legend: $s_{1}$—waist, $s_{2}$—chest, $s_{3}$—leg, $s_{4}$—arm.

## Data Availability

A publicly available dataset was analyzed in this study. This data can be found here: https://ibigworld.ath.edu.pl/index.php/en/ (accessed on 4 November 2021).

## Abbreviations

The following abbreviations are used in this manuscript:

*t*	time step
$D_{i}^{s}$	set of activities for which *i*-th sensor node should send $d_{i,t}$ to the main node
*D*	set of all recognized activities (activities are denoted by natural numbers, $D_{i}^{s}\subset D$)
$d_{i,t}$	activity recognized by sensor node *i* at time step *t*
$d_{t}$	activity recognized by the main node at time step *t* based on the voting procedure
$w(d_{i,t})$	weight of the recognized activity ($w\left(d_{i,t}\right)\in \left[0,1\right]$)
$s_{i,t}$	binary variable, which describes the necessity of sending $d_{i,t}$ to the main node
$M_{i}$	machine learning model used by *i*-th sensor node
$X_{i}\left(t\right)$	time series of sensor readings collected by node *i* at time step *t*
a	accuracy of activity recognition
ϵ	tuning parameter, which helps to neglect the influence of outliers

## Appendix A. Verification of a Model

The proposed method was verified for trained models and predefined set *D* according to Table 1. The various sensor configuration results were evaluated using basic measures [55] . as: True Positives, False Positives, True Negative, False Negative, Recall, Precision, Sensitivity, Specificity, F-means, Accuracy (Acc) and Cohen’s cappa. The detailed results for particular configurations from Figure 8 are presented in Table A1,Table A2,Table A3 and Table A4.

**Table A1:** The activity classification results for sensor $s_{1}$.

	True Positives	False Positives	True Negative	False Negative	Recall	Precision	Sensivity	Specificity	F-means	Accuracy	Cohen’s Cappa
1	299	38	2517	66	82%	89%	82%	99%	85%		
2	363	34	2521	2	99%	91%	99%	99%	95%		
3	364	13	2542	1	100%	97%	100%	99%	98%		
4	358	8	2547	7	98%	98%	98%	100%	98%		
5	351	119	2436	14	96%	75%	96%	95%	84%		
6	246	22	2533	119	67%	92%	67%	99%	78%		
7	304	30	2525	61	83%	91%	83%	99%	87%		
8	365	6	2549	0	100%	98%	100%	100%	99%		
Total										91%	89%

**Table A2:** The activity classification results for sensor $s_{1}$ and $s_{3}$ as support nodes.

	True Positives	False Positives	True Negative	False Negative	Recall	Precision	Sensivity	Specificity	F-means	Accuracy	Cohen’s Cappa
1	299	3	2552	66	82%	99%	82%	100%	90%		
2	363	31	2524	2	99%	92%	99%	99%	96%		
3	364	0	2555	1	100%	100%	100%	100%	100%		
4	358	1	2554	7	98%	100%	98%	100%	99%		
5	335	26	2529	30	92%	93%	92%	99%	92%		
6	350	11	2544	15	96%	97%	96%	100%	96%		
7	364	49	2506	1	100%	88%	100%	98%	94%		
8	365	1	2554	0	100%	100%	100%	100%	100%		
Total										96%	95%

**Table A3:** The activity classification results for sensor $s_{4}$.

	True Positives	False Positives	True Negative	False Negative	Recall	Precision	Sensivity	Specificity	F-means	Accuracy	Cohen’s Cappa
1	284	28	2527	81	78%	91%	78%	99%	84%		
2	351	3	2552	14	96%	99%	96%	100%	98%		
3	273	195	2360	92	75%	58%	75%	92%	66%		
4	298	42	2513	67	82%	88%	82%	98%	85%		
5	365	15	2540	0	100%	96%	100%	99%	98%		
6	306	91	2464	59	84%	77%	84%	96%	80%		
7	321	37	2518	44	88%	90%	88%	99%	89%		
8	310	1	2554	55	85%	100%	85%	100%	92%		
Total										86%	84%

**Table A4:** The activity classification results for sensor $s_{4}$; and $s_{1}$, $s_{2}$, and $s_{3}$ as support nodes.

	True Positives	False Positives	True Negative	False Negative	Recall	Precision	Sensivity	Specificity	F-means	Accuracy	Cohen’s Cappa
1	348	9	2546	17	95%	97%	95%	100%	96%		
2	365	4	2551	0	100%	99%	100%	100%	99%		
3	361	101	2454	4	99%	78%	99%	96%	87%		
4	268	1	2554	97	73%	100%	73%	100%	85%		
5	362	1	2554	3	99%	100%	99%	100%	99%		
6	359	10	2545	6	98%	97%	98%	100%	98%		
7	363	4	2551	2	99%	99%	99%	100%	99%		
8	360	4	2551	5	99%	99%	99%	100%	99%		
Total										95%	95%
